# Changes in Staging and Management of Non-Small Cell Lung Cancer (NSCLC) Patients Following the Implementation of Low-Dose Chest Computed Tomography (LDCT) Screening at Kaohsiung Medical University Hospital

**DOI:** 10.3390/cancers16223727

**Published:** 2024-11-05

**Authors:** Chin-Ling Chen, Jui-Sheng Hsu, Yi-Wen Shen, Chih-Hsiang Hsu, Shih-Yu Kao, Wei-An Lai, Cheng-Hao Chuang, Yu-Wei Liu, Jui-Ying Lee, Shah-Hwa Chou, Jen-Yu Hung, Inn-Wen Chong, Chih-Jen Yang

**Affiliations:** 1 Cancer Center, Kaohsiung Medical University Hospital, Kaohsiung Medical University, Kaohsiung 80708, Taiwan; 960645@kmuh.org.tw (C.-L.C.); iwen69228@hotmail.com (Y.-W.S.); luluadrian0592@gmail.com (C.-H.H.); scyy3266@gmail.com (S.-Y.K.); 2 Department of Nursing, Kaohsiung Kaohsiung Medical University, Kaohsiung 80708, Taiwan; 3 Department of Radiology, Kaohsiung Medical University Hospital, Kaohsiung Medical University, Kaohsiung 80708, Taiwan; jshsu@kmu.edu.tw; 4 Department of Pathology, Kaohsiung Kaohsiung Medical University, Kaohsiung 80708, Taiwan; weian930@gmail.com; 5 Division of Pulmonary and Critical Care Medicine, Department of Internal Medicine, Kaohsiung Medical University Hospital, Kaohsiung Medical University, Kaohsiung 80708, Taiwan; aeafish@gmail.com (C.-H.C.); jenyuhung@gmail.com (J.-Y.H.); 6 Graduate Institute of Medicine, College of Medicine, Kaohsiung Medical University, Kaohsiung 80708, Taiwan; 7 Division of Thoracic Surgery, Department of Surgery, Kaohsiung Medical University Hospital, Kaohsiung Medical University, Kaohsiung 80708, Taiwan; nipma6714@gmail.com (Y.-W.L.); rockwell0111@gmail.com (J.-Y.L.); shhwch@cc.kmu.edu.tw (S.-H.C.); 8 Graduate Institute of Clinical Medicine, College of Medicine, Kaohsiung Medical University, Kaohsiung 80708, Taiwan; 9 School of Post-Baccalaureate Medicine, College of Medicine, Kaohsiung Medical University, Kaohsiung 80708, Taiwan

**Keywords:** non-small cell lung cancer, low-dose computed tomography, LDCT, lung cancer staging, early detection, five-year survival, adenocarcinoma, squamous cell carcinoma

## Abstract

Background: Low-dose computed tomography (LDCT) has been widely adopted for lung cancer screening due to its proven ability to reduce lung cancer mortality, especially among high-risk populations. Methods: This retrospective study aims to evaluate the impact of LDCT screening on non-small cell lung cancer (NSCLC) staging at Kaohsiung Medical University Hospital (KMUH) from 2011 to 2020, following the introduction of LDCT in 2013. The study examines the correlation between LDCT screening volume and changes in the distribution of NSCLC stages, particularly early-stage (stages 0 and I) and late-stage (stage IV) diagnoses. Additionally, it explores the differences in histopathological subtypes, focusing on adenocarcinoma and squamous cell carcinoma, and assesses the impact of early detection on five-year survival rates. Results: The results show a significant increase in early-stage NSCLC diagnoses, particularly in adenocarcinoma cases, where early-stage diagnoses rose from 10.4% in 2010 to 38.7% in 2019. However, the number of stage IV cases remained stable, indicating that LDCT may not substantially reduce late-stage diagnoses. Pearson’s correlation analysis demonstrated a strong positive correlation between LDCT screening and early-stage NSCLC detection, particularly for adenocarcinoma (*p* < 0.001), though the early detection of squamous cell carcinoma and small cell carcinoma remained limited. Conclusions: The study concludes that LDCT screening plays a crucial role in improving early NSCLC detection and five-year survival rates. Future research should focus on optimizing screening strategies to capture more at-risk populations and enhance the detection of harder-to-diagnose subtypes like squamous cell carcinoma.

## Simple Summary

Since the U.S. National Lung Screening Trial (NLST) demonstrated the efficacy of low-dose computed tomography (LDCT) for early lung cancer detection, LDCT has emerged as a critical tool for identifying lung cancer in high-risk populations. This retrospective study evaluates trends in non-small cell lung cancer (NSCLC) staging at Kaohsiung Medical University Hospital (KMUH) from 2011 to 2020, with a particular focus on the effects of LDCT screening, which was introduced in 2013. We examined correlations between the number of LDCT screenings and NSCLC stage distribution, emphasizing early-stage (stages 0 and I) and late-stage (stage IV) diagnoses. Additionally, we assessed histopathological differences between adenocarcinoma and squamous cell carcinoma identified via LDCT and evaluated the impact of early diagnosis on five-year survival rates.

## 1. Introduction

Lung cancer remains the leading cause of cancer-related mortality worldwide, accounting for nearly 1.8 million deaths annually, with non-small cell lung cancer (NSCLC) representing approximately 85% of cases [1]. The poor prognosis for lung cancer patients is primarily due to the fact that most cases are diagnosed at an advanced stage, where curative treatment options are limited. In Taiwan, the incidence rates of lung adenocarcinoma in both sexes increased from 1997 to 2017 [2], whereas those of lung squamous cell carcinoma decreased. Early detection is widely recognized as a key strategy in improving survival outcomes, particularly for NSCLC, where early-stage diagnosis offers significantly better prognosis [3,4]. Low-dose computed tomography (LDCT) is designed specifically to reduce radiation exposure while maintaining sufficient image quality to detect early-stage lung cancer. The radiation dose in LDCT is significantly lower than that of a standard diagnostic CT scan, which makes it safer for long-term screening, especially for high-risk populations like smokers who may need repeated scans. LDCT uses only about 20–25% of the radiation dose used in a standard CT scan. While a conventional chest CT scan typically exposes a patient to around 7–8 millisieverts (mSv) of radiation, LDCT reduces this to about 1–1.5 mSv [5]. This dose reduction helps minimize the cumulative radiation risk, which is particularly important for individuals undergoing regular screening. The lower radiation dose in LDCT is especially advantageous for lung cancer screening programs, where high-risk individuals may require annual screening over many years. By significantly reducing radiation exposure, LDCT minimizes the potential risks associated with repeated scans, such as the increased likelihood of radiation-induced cancers over time. Low-dose computed tomography (LDCT) has emerged as a crucial tool in lung cancer screening, particularly for high-risk populations such as smokers, as demonstrated in several landmark studies. The National Lung Screening Trial (NLST) was a pivotal randomized controlled trial in the U.S. that showed a 20% reduction in lung cancer mortality among high-risk smokers screened with LDCT compared to chest X-ray [5]. Similarly, the NELSON trial, conducted in Europe, further confirmed LDCT’s effectiveness, reporting a 24% reduction in lung cancer mortality in men and an even higher reduction of 33% in women [6]. In Taiwan, the TALENT trial (Taiwan Lung Cancer Screening in Never-Smokers) focused on a different demographic—never-smokers—and demonstrated that LDCT screening can be beneficial for early lung cancer detection even in populations where smoking is not the primary risk factor [7]. This trial highlighted the relevance of LDCT screening in non-Western populations, such as Taiwanese, where environmental and genetic factors might play a more significant role in lung cancer development. On the European front, the European Position Statement on Lung Cancer Screening advocates for implementing LDCT screening based on the successes of trials like NLST and NELSON, emphasizing its importance in reducing mortality in high-risk groups [8]. Furthermore, the International Association for the Study of Lung Cancer (IASLC) supports the global implementation of LDCT screening for high-risk populations, underscoring its role in decreasing lung cancer-related deaths [9]. Additionally, long-term follow-up studies, such as the NLST’s cost-effectiveness analysis, confirm that LDCT screening is not only life-saving but also cost-effective for high-risk groups, further supporting its adoption in clinical practice [10]. Kaohsiung Medical University Hospital (KMUH), an 1800-bed tertiary medical center in Taiwan, introduced LDCT screening as part of health exams in 2013. This study aims to evaluate the impact of LDCT on non-small cell lung cancer (NSCLC) staging trends at KMUH from 2011 to 2020, with a focus on shifts in early- and late-stage diagnoses. Additionally, we assess the relationship between LDCT screening and histopathological differences in adenocarcinoma and squamous cell carcinoma and analyze how early-stage detection affects five-year survival rates.

## 2. Materials and Methods

### 2.1. Study Design and Data Collection

This retrospective cohort study utilized data from Kaohsiung Medical University Hospital (KMUH), including LDCT screening records and NSCLC staging information from 2011 to 2020. Data from the Taiwan National Cancer Registry through 2022 were also incorporated to ensure accurate staging and survival outcomes. KMUH initiated LDCT screening in 2013, and data collection includes the total number of LDCT screenings performed annually and the corresponding number of new NSCLC diagnoses by stage (stages 0, I, II, III, and IV).

Patient records were reviewed to classify tumors according to TNM staging based on the eighth edition of the American Joint Committee on Cancer (AJCC) staging system. Histopathological data were extracted to identify cases of adenocarcinoma and squamous cell carcinoma, the two most common NSCLC subtypes. Data were aggregated by year to evaluate staging trends, histopathological findings, and survival outcomes.

### 2.2. Statistical Analysis

Statistical analyses were performed using Pearson’s correlation coefficients to assess the relationships between LDCT screening volume and NSCLC stage distribution. *p*-values were calculated to evaluate the significance of observed correlations, with a threshold for statistical significance set at *p* < 0.05. We further analyzed trends in early-stage (stages 0 and I) and late-stage (stage IV) diagnoses in relation to the increase in LDCT screening. To assess survival outcomes, all patients were followed up to 31 December 2022. Kaplan–Meier survival curves were generated for five-year overall survival rates and trends of five-year survival rates in different stages for NSCLC patients at KMUH was also demonstrated.

## 3. Results

### 3.1. Increase in LDCT Screening Volume

**Figure 1:**
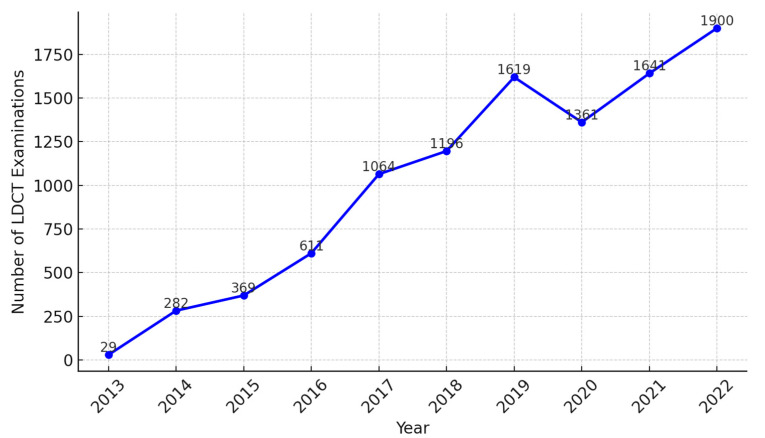
LDCT examination over years 2013–2022.

From 2013, when LDCT screening was first implemented at KMUH, until December 2022, there was a substantial increase in the number of screenings performed. In 2013, only 29 LDCT screenings were conducted, but by 2022, this number had risen to 1900. The Pearson correlation coefficient between the number of LDCT screenings and year was 0.98 (*p* = 1.56 × 10^−6^), indicating a strong positive correlation (Figure 1). This sharp increase in screening volume reflects growing awareness of the benefits of early detection among high-risk populations and highlights the commitment of KMUH to expanding lung cancer screening initiatives.

### 3.2. The Detection Rate of Lung Cancer by LDCT at KMUH (2013–2022)

**Figure 2:**
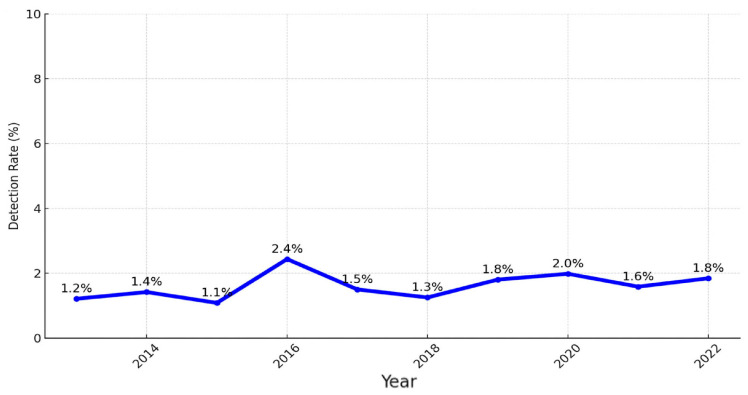
The detection rate of lung cancer by LDCT at KMUH (2013–2022).

The detection rate of lung cancer by LDCT is defined as the number of newly diagnosed lung cancer patients divided by the total number of LDCT scans performed at KMUH (Figure 2). In 2013, the detection rate was 1.2%, with the highest rate recorded at 2.4% in 2016. Notably, patients diagnosed outside of KMUH were excluded from this analysis.

#### 3.2.1. The Distribution Stages of Lung Cancer by LDCT at KMUH (2013–2022)

**Figure 3:**
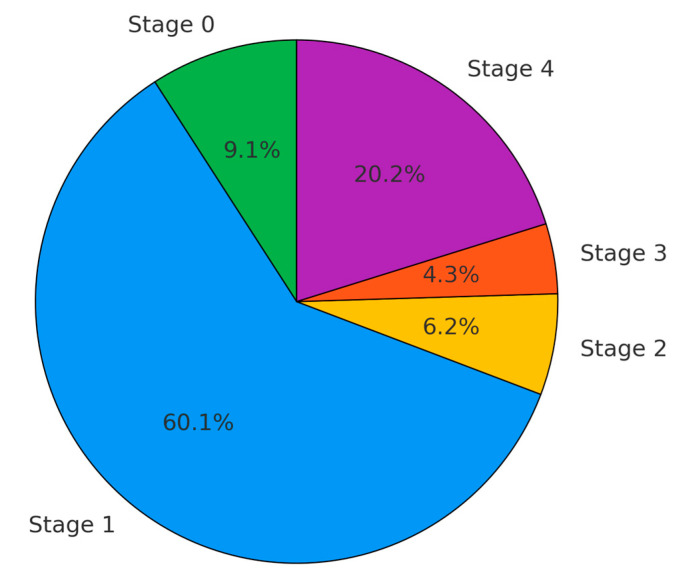
Stage distribution of all LDCT screen lung cancer by LDCT at KMUH (2013 to 2022).

In the LDCT-diagnosed lung cancer cohort (Figure 3), stage I was the most common, accounting for 60.1% of cases. Additionally, stage 0 represented 9.1%, stage II accounted for 6.2%, stage III for 4.3%, and stage IV for 20.2%. Overall, approximately 80% of cases detected by LDCT were in early or locally advanced stages, offering the potential for curative treatment through surgical intervention.

#### 3.2.2. The Percentage of Each Stages Underwent Operation in the LDCT-Diagnosed Lung Cancer Cohort

**Figure 4:**
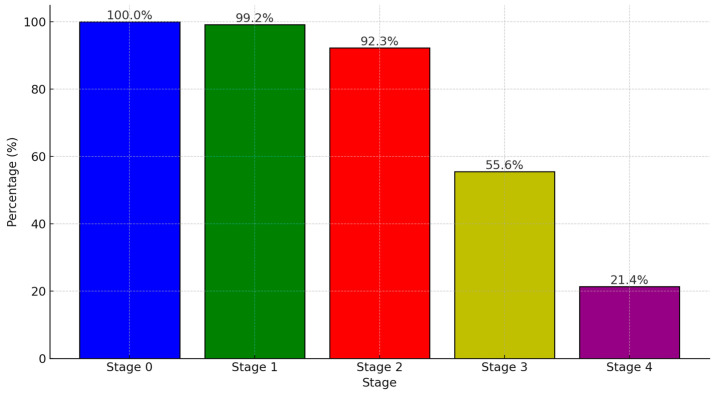
The percentage of patients at each stage who underwent operation in the LDCT-diagnosed lung cancer cohort in KMUH (2013 to 2022).

In the LDCT-diagnosed lung cancer cohort (Figure 4), the majority of patients underwent surgical intervention. All stage 0 patients (100%) underwent surgery, followed by 99.2% of stage I patients, 92.3% of stage II patients, 55.6% of stage III patients, and 21.4% of stage IV patients.

### 3.3. Shifts in NSCLC Stage Distribution in All NSCLC in KMUH from 2011 to 2020

#### 3.3.1. Early-Stage Diagnoses (Stages 0 and I)

**Figure 5:**
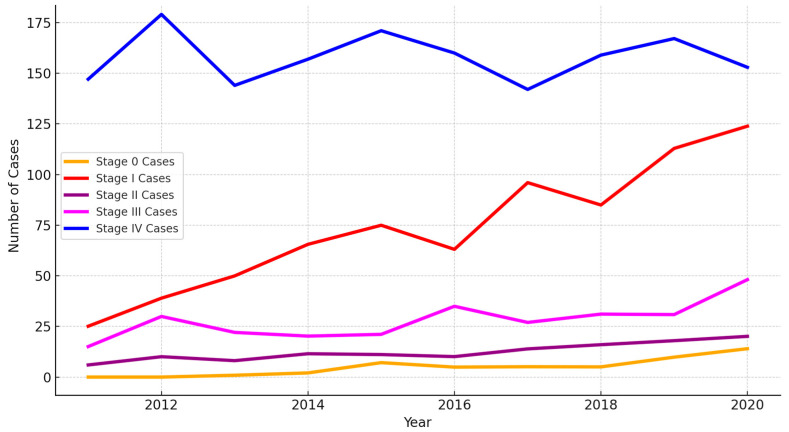
The trends in NSCLC patients by stage (2011–2020) in KMUH.

**Figure 6:**
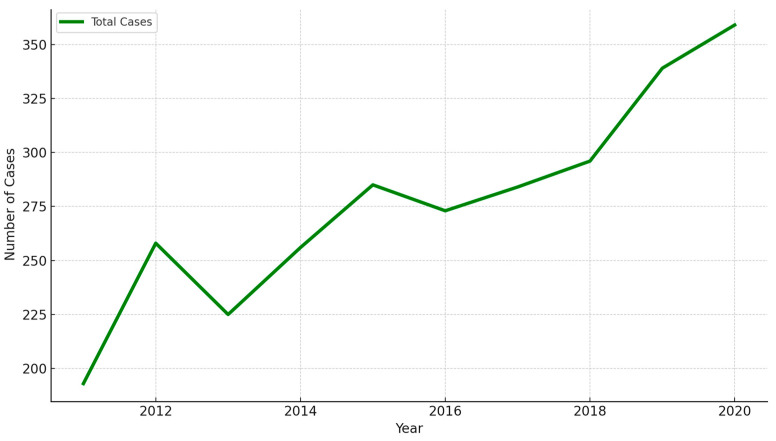
The trend of total newly diagnosed NSCLC cases in KMUH (2011–2020).

The number of early-stage NSCLC cases (stages 0 and I) rose significantly from 25 in 2011 to 138 in 2020. Pearson’s correlation coefficient was 0.97 (*p* = 5.62 × 10^−6^), indicating a statistically significant increase in early-stage diagnoses (Figure 5). This trend reflects the effectiveness of LDCT in detecting early-stage tumors, which are often asymptomatic and undetectable on standard radiographs and the annually new NSCLC cases also elevated (Figure 6).

#### 3.3.2. Stage II Diagnoses

The number of stage II cases increased from 6 in 2011 to 20 in 2020, with a Pearson correlation coefficient of 0.94 (*p* = 5.23 × 10^−5^). This statistically significant increase suggests that LDCT also contributes to identifying cancers at an intermediate stage, enabling earlier intervention.

#### 3.3.3. Stage III Diagnoses

Stage III diagnoses fluctuated over the study period but showed an overall upward trend, increasing from 15 in 2011 to 48 in 2020. The Pearson correlation coefficient was 0.75 (*p* = 0.012), indicating moderate statistical significance. These fluctuations may reflect variations in patient presentation and disease progression, particularly among those who present with symptoms after an initial diagnosis.

#### 3.3.4. Stage IV Diagnoses

Interestingly, the number of stage IV diagnoses remained relatively stable throughout the study period, increasing only slightly from 147 cases in 2011 to 153 in 2020. Pearson’s correlation coefficient was -0.02 (*p* = 0.9609), suggesting no significant linear correlation between the increase in LDCT screening and late-stage diagnoses. This finding indicates that while LDCT is effective in detecting early-stage NSCLC, additional interventions are necessary to address late-stage cancer, which may be less amenable to early detection due to the aggressiveness of certain tumors or delays in seeking medical attention.

The statistical analysis of the trend in total NSCLC cases from 2011 to 2020 reveals the R-squared value of 0.87 indicates a strong correlation between the year and the increase in case numbers (*p* < 0.001).

### 3.4. Histopathological Findings

#### 3.4.1. Adenocarcinoma

**Figure 7:**
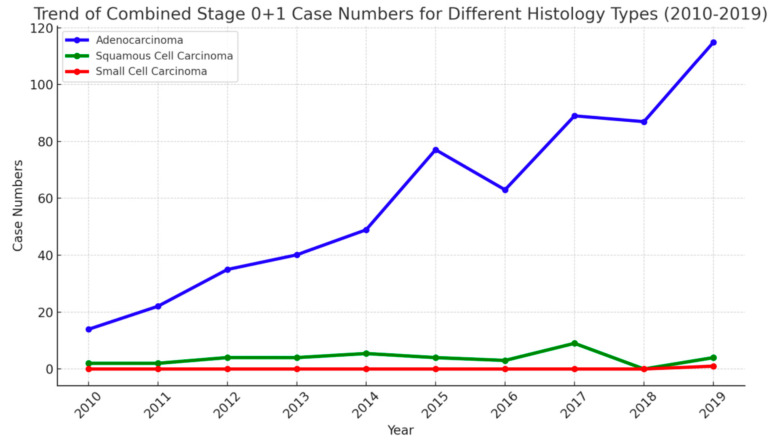
The trends in early-stage NSCLC and SCLC by cancer type (2010–2019) in KMUH.

Early-stage adenocarcinoma cases (stages 0 and I) increased dramatically from 2010 to 2019. The Pearson correlation coefficient was 0.97 (*p* = 5.62 × 10^−6^), underscoring the strong positive association between LDCT screening and the detection of early-stage adenocarcinoma (Figure 7). Given that adenocarcinoma is the most common NSCLC subtype, this result demonstrates the crucial role of LDCT in identifying early-stage tumors with favorable prognoses.

#### 3.4.2. Squamous Cell Carcinoma

In contrast, early-stage squamous cell carcinoma (stages 0 and I) showed a modest increase, rising from two cases in 2010 to four cases in 2019. The Pearson correlation coefficient was 0.52 (*p* = 0.12), suggesting a moderate correlation but without statistical significance. This discrepancy may reflect differences in tumor biology between adenocarcinoma and squamous cell carcinoma, with the latter often presenting later due to its central location and propensity for symptom onset in earlier stages.

#### 3.4.3. Small Cell Carcinoma

The number of early-stage small cell lung cancer (SCLC) patients (stage I) increased slightly, from zero patients (0%) in 2010 to one patient (8.3%) in 2019. The Pearson correlation coefficient was 0.52, with a *p*-value of 0.121, indicating a moderate correlation between the number of LDCT screenings and early-stage SCLC diagnoses, but this correlation was not statistically significant.

### 3.5. The Role of Surgery as the First-Line Treatment

**Figure 8:**
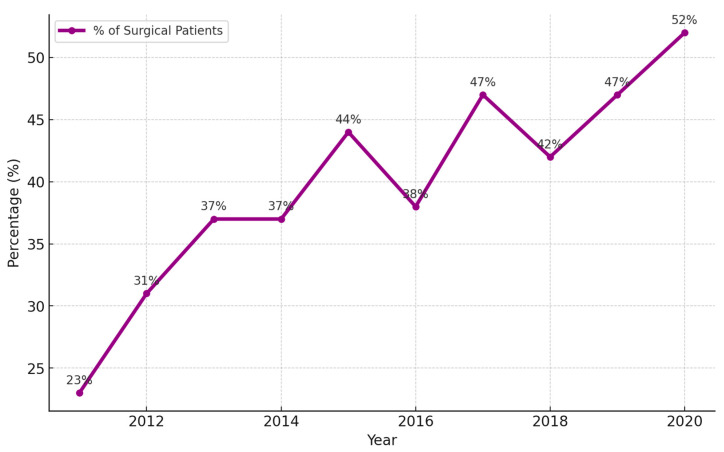
The trend of the percentage of NSCLC patients receiving surgery as the first-line treatment in KMUH.

In Figure 8, the trend demonstrates a notable increase in the percentage of NSCLC patients receiving surgery as the first-line treatment, rising significantly over the decade from 23.0% to 52% in 2020. This upward trend underscores the critical role of surgery in managing NSCLC, particularly in early-stage patients where surgical intervention offers the best chance for long-term survival. As more patients are diagnosed at earlier stages, the choice of surgery as the primary treatment option becomes increasingly vital for improving outcomes and survival rates in NSCLC care.

### 3.6. Impact on Five-Year Survival Rates

**Figure 9:**
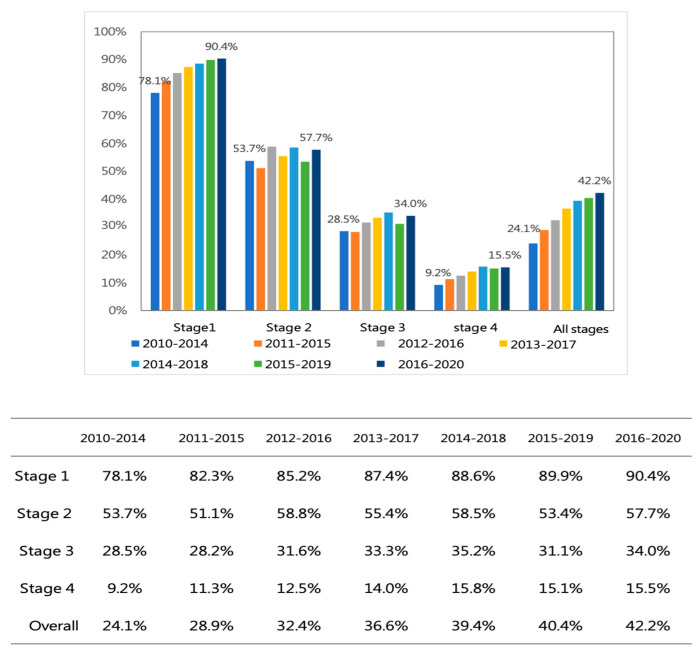
The trends of five-year survival rates in different stages for NSCLC patients at KMUH.

Five-year survival rates for NSCLC patients at KMUH improved across all stages between 2010 and 2020. The five-year survival rate for stage I patients consistently exceeded 80%, while stage IV and overall stage patients also showed improved outcomes (Figure 9).

## 4. Discussion

This study highlights the significant impact of low-dose computed tomography (LDCT) on the stage distribution of non-small cell lung cancer (NSCLC) at KMUH, particularly in increasing early-stage diagnoses. The rise in early-stage diagnoses, especially among adenocarcinoma patients, confirms LDCT’s effectiveness in detecting asymptomatic tumors with the best prognosis. These findings align with international research, establishing LDCT as a crucial tool for lung cancer screening. By minimizing radiation exposure compared to standard CT, LDCT reduces risks such as radiation-induced cancers, making it suitable for repeated screenings.

While LDCT reduces lung cancer mortality in high-risk populations, challenges remain, including false positives, overdiagnosis, and incidental findings [11,12,13,14]. Most studies did not use current nodule evaluation protocols, which could reduce unnecessary procedures. Additionally, economic constraints and a lack of government programs in some Asian regions hinder widespread LDCT implementation, though strategies have been suggested to overcome these barriers [15].

In Taiwan, an analysis of national data from 1994 to 2020 revealed an increase in early-stage diagnoses, improving five-year survival rates from 22.1% to 54.9%. Despite this, stage IV diagnoses remained stable, indicating that LDCT may not fully reduce late-stage incidence in all populations. Factors such as rapid progression of late-stage cancer and limitations in screening criteria may contribute to this [3].

LDCT proved especially effective for early-stage adenocarcinoma detection, while squamous cell carcinoma showed only modest improvements, suggesting the need for alternative screening approaches. Improvements in five-year survival rates across all stages, particularly in stage I patients, emphasize the life-saving potential of early detection. Even advanced-stage patients saw survival gains, likely due to advancements in treatments like targeted therapies and immunotherapy [1,3].

However, LDCT has limitations, such as high false-positive rates, overdiagnosis, and radiation exposure from repeated screenings [7,12,16]. Screening high-risk individuals with LDCT can reduce lung cancer mortality but may also result in false-positive findings, leading to unnecessary tests and invasive procedures, overdiagnosis, incidental findings, increased patient distress, and, in rare cases, radiation-induced cancers [17,18,19]. Overdiagnosis can lead to unnecessary treatments, and the psychological and financial burdens of follow-up testing are significant. While LDCT is beneficial for high-risk groups, its use in lower-risk populations, like never-smokers, is debated, with limited data on its cost-effectiveness and overall benefit [11,20,21].

In addition to smoking, air pollution, particularly PM2.5, is a significant lung cancer risk factor [22,23,24,25,26]. PM2.5 exposure was responsible for 223,000 lung cancer deaths globally in 2010, with over half in East Asia. Studies link long-term PM2.5 exposure to higher lung cancer mortality in never-smokers. In Taiwan, worsening air quality in Southern Taiwan correlated with lower survival rates in adenocarcinoma patients compared to Northern Taiwan. PM2.5 also promotes tumorigenesis through inflammation, highlighting the need for policies addressing air pollution to mitigate lung cancer risk [26,27].

## 5. Clinical Implications

The results of this study have important clinical implications for lung cancer screening and management. First, the findings support expanding LDCT screening programs, especially in regions with high smoking rates or environmental risk factors. The strong correlation between LDCT screening volume and early-stage NSCLC detection suggests that increasing access to LDCT could lead to earlier diagnoses and improved survival outcomes. Given LDCT’s success in the early detection of adenocarcinoma, it should be considered a standard of care for high-risk populations, with efforts to enhance accessibility.

However, the relatively modest increase in early-stage squamous cell carcinoma detection indicates a need for further research into complementary screening modalities.

PET has been widely studied for diagnosing indeterminate lung lesions and has proven to be significantly more accurate than CT in distinguishing between benign and malignant lesions as small as 1 cm. PET has an overall sensitivity of 96%, a specificity of 79%, and an accuracy of 91%. However, false negatives may occur in lesions smaller than 1 cm, as PET requires a sufficient mass of metabolically active malignant cells. As a result, PET is not suitable for detecting early-stage squamous cell carcinoma of the lung. Additionally, false positives can occur in cases of inflammation or granulomatous disease [28,29,30,31].

Future studies should explore whether additional molecular biomarkers could improve early detection rates for squamous cell carcinoma. Refining LDCT screening criteria to capture more at-risk individuals, including non-smokers or those exposed to environmental carcinogens, is another area for exploration.

## 6. Limitations

While this study provides valuable insights into the impact of LDCT screening on NSCLC staging, several limitations should be noted. First, as a retrospective analysis, it is subject to potential biases in data collection and reporting. Second, not all patients with positive findings in LDCT who underwent surgery were screened at KMUH; some may have been screened by LDCT or treated at other hospitals. Additionally, although KMUH is a large medical center in Taiwan, the study’s findings are based on data from a single institution, which may limit the generalizability to other regions or healthcare systems. Future research should incorporate multi-center or population-based approaches to further validate these results.

Another limitation is the lack of comprehensive smoking or family history data for all patients, which may influence the interpretation of the screening outcomes. Smoking is a major risk factor for NSCLC, and the absence of detailed smoking history could confound the relationship between LDCT screening and cancer staging. More comprehensive data on smoking exposure, as well as other risk factors like family history and occupational exposures, would enhance the strength of future analyses.

Finally, this study did not examine the cost-effectiveness of implementing widespread LDCT programs. Although LDCT has been shown to reduce lung cancer mortality, its cost-effectiveness depends on several factors, including the cost of screening, the frequency of false positives, and the subsequent use of diagnostic procedures. Future research should assess the economic implications of LDCT screening to inform healthcare policy decisions and optimize resource allocation.

## 7. Future Directions

Building on the findings of this study, future research should focus on several key areas. First, optimizing LDCT screening protocols, particularly in relation to screening frequency, is critical to maximize early detection while minimizing unnecessary radiation exposure and false-positive results. Additionally, studies should investigate the potential role of genetic and molecular biomarkers in identifying individuals at high risk for lung cancer, including those who do not meet current screening guidelines based solely on smoking history.

Moreover, given the relatively stable rate of stage IV diagnoses, future research should focus on developing targeted interventions for patients at risk of advanced lung cancer. This could include integrating molecular profiling and personalized medicine approaches to better understand the biological mechanisms driving tumor progression and treatment resistance. Clinical trials that evaluate the combination of LDCT with novel imaging technologies or blood-based biomarkers could provide insights into improving early detection, especially for challenging subtypes like squamous cell carcinoma.

As immunotherapy and targeted therapies continue to advance, future studies should also assess how early-stage diagnoses through LDCT influence treatment outcomes in NSCLC patients. Understanding the relationship between early detection and newer therapeutic strategies will be crucial for improving long-term survival and quality of life for lung cancer patients.

## 8. Conclusions

This study underscores the pivotal role of LDCT in the early detection and staging of NSCLC, particularly in identifying early-stage adenocarcinoma. The strong correlation between LDCT screening volume and early-stage NSCLC diagnoses highlights the life-saving potential of widespread screening in high-risk populations. While LDCT has proven effective in improving early detection and survival rates, challenges remain in detecting certain subtypes, such as squamous cell carcinoma, and in reducing late-stage diagnoses.

The findings suggest that future efforts should focus on refining screening strategies, expanding screening eligibility, and integrating additional diagnostic tools to improve outcomes for all lung cancer patients. By prioritizing early detection and personalized interventions, LDCT screening has the potential to significantly reduce lung cancer mortality and enhance public health outcomes.

## Data Availability

Data are contained within the article.
